# Photostability of commercial sunscreens upon sun exposure and irradiation by ultraviolet lamps

**DOI:** 10.1186/1471-5945-7-1

**Published:** 2007-02-26

**Authors:** Helena Gonzalez, Nils Tarras-Wahlberg, Birgitta Strömdahl, Asta Juzeniene, Johan Moan, Olle Larkö, Arne Rosén, Ann-Marie Wennberg

**Affiliations:** 1 Department of Dermatology, Sahlgrenska University Hospital, SE-413 45 Göteborg, Sweden; 2 Department of Physics, Göteborg University, SE-412 96 Göteborg, Sweden; 3 Department of Radiation Biology, Institute for Cancer Research, The Norwegian Radium Hospital, Montebello, Oslo, N-0310, Norway

## Abstract

**Background:**

Sunscreens are being widely used to reduce exposure to harmful ultraviolet (UV) radiation. The fact that some sunscreens are photounstable has been known for many years. Since the UV-absorbing ingredients of sunscreens may be photounstable, especially in the long wavelength region, it is of great interest to determine their degradation during exposure to UV radiation. Our aim was to investigate the photostability of seven commercial sunscreen products after natural UV exposure (UVnat) and artificial UV exposure (UVart).

**Methods:**

Seven commercial sunscreens were studied with absorption spectroscopy. Sunscreen product, 0.5 mg/cm^2^, was placed between plates of silica. The area under the curve (AUC) in the spectrum was calculated for UVA (320–400 nm), UVA1 (340–400 nm), UVA2 (320–340 nm) and UVB (290–320 nm) before (AUC_before_) and after (AUC_after_) UVart (980 kJ/m^2 ^UVA and 12 kJ/m^2 ^of UVB) and before and after UVnat. If theAUC Index (AUCI), defined as AUCI = AUC_after_/AUC_before_, was > 0.80, the sunscreen was considered photostable.

**Results:**

Three sunscreens were unstable after 90 min of UVnat; in the UVA range the AUCI was between 0.41 and 0.76. In the UVB range one of these sunscreens was unstable with an AUCI of 0.75 after 90 min. Three sunscreens were photostable after 120 min of UVnat; in the UVA range the AUCI was between 0.85 and 0.99 and in the UVB range between 0.92 and 1.0. One sunscreen showed in the UVA range an AUCI of 0.87 after UVnat but an AUCI of 0.72 after UVart. Five of the sunscreens were stable in the UVB region.

**Conclusion:**

The present study shows that several sunscreens are photounstable in the UVA range after UVnat and UVart. There is a need for a standardized method to measure photostability, and the photostability should be marked on the sunscreen product.

## Background

Sunscreens give good protection against sunburn, actinic keratosis and squamous cell carcinoma. The results for preventing cutaneous malignant melanoma (CMM) and basal cell carcinoma are less conclusive [1-3]. One explanation for this can be that UVA radiation (320–400 nm) plays a role for induction of CMM [4] and that it is mainly in the UVA range the photodegradation of the sunscreen occurs. In the present work, commercially available sunscreens, containing organic chemical and/or inorganic chemical filters, have been exposed to natural UV (UVnat) as well as to artificial UV (UVart) in order to study their photostability.

Previous studies have shown that some sunscreens lose part of their protection when exposed to UV radiation [5-10]. Several sunscreen producers claim that their products give good protection against both UVA and UVB radiation; however, the photostability of the product is rarely declared. This is also important for the consumer to know when choosing a sunscreen. Since it has been known for several years that some products may be photounstable, one would have expected a large improvement in the photostability of sunscreen products. Up to now, there is no standard method for determining photostability of a sunscreen [6,11,12]. Neither is there an international standard method for measuring UVA protection, and several different systems are currently in use [13-16].

The aim of this study was to investigate the photostability of commercial sunscreen products after UVnat and after UVart.

## Methods

### Sunscreens

Seven commercial sunscreens were included, all available on the Swedish market. Three sunscreens contained only organic chemical filters, three sunscreens had a combination of inorganic and organic chemical filters, and one sunscreen contained solely inorganic chemical filters. In Table 1 the photoactive compounds of the sunscreens and the Sun Protection Factor (SPF) of the product are shown.

The sunscreen was weighed and placed between two plates of polished fused silica (quartz) with diameter 25 mm and thickness 5 mm. The amount applied was 0.5 mg/cm^2^. The absorbance was too high for proper measurements when the recommended amount of 2 mg/cm^2 ^was applied, causing distortion in the absorption spectrum. For this reason a thinner layer was applied. A previous study has shown that the result were independent whether an application thickness of 1 or 2 mg/cm^2 ^was used [17].

### Light sources

For UVA radiation, a UVASUN 2000 (MUTZHAS, Germany) was used. The output is mainly between 340 and 400 nm.

For UV radiation (including UVB), an Esshå Corona Mini (Sweden), equipped with two fluorescent tubes, Philips TL 12 (20 W), was used. This is a broadband radiation source from 280 to 380 nm with a major peak at 313 nm. There are strong mercury peaks at 313 nm and 365 nm.

The irradiance at the exposure plane was measured with an International Light IL 1350 Radiometer/Photometer using a probe named SED 240 for UVA and a probe named SED 015 for UVB radiation. The fluence rate of the UVA lamp was 820 W/m^2 ^when measured from a distance of 25 cm. Twenty minutes' exposure gave a dose of 980 kJ/m^2^. This corresponds to the UVA dose that reaches the earth's surface during one sunny summer day in Gothenburg [18]. We also measured the spectral distribution of the UVB lamp. By combining the spectral distribution with the action spectrum of the probe, the fluence rate of the UVB radiation was 9.8 W/m^2^. Twenty minutes of exposure gave a dose of 12 kJ/m^2 ^UV radiation (including UVB). This corresponds to 45 Standard Erythema Doses (SED) when further weighted by the CIE action spectrum [19]. This is a much higher dose than normal for one summer day in Gothenburg [18] or what has been reported from Denmark [20]. In spite of that fact, the majority of sunscreens showed good stability in the UVB range. In Table 2 the UV doses reported from the Swedish Metrological and Hydrological Institute (SMHI) are listed.

For UVnat, samples were placed horizontally outdoors when the weather was sunny. This was done in early July in Gothenburg (latitude: 57° 42' N). The total exposure time was 120 min (Table 2) with measurements of the absorption spectra before exposure and after 30 min, 90 min and 120 min of UVnat. SMHI measures the global irradiance in many places in Sweden and gives the CIE erythema weighted UV radiation as well (Table 2).

To eliminate the possibility that the degradation of the photoactive compounds could be caused by a temperature increase, control samples of sunscreen between silica plates were placed on a heating plate for 20 minutes. The temperature was kept at 50°C ± 2°C, which was the same as that measured during exposure to the UVA lamp. This is about 15°C higher than the temperature of the skin. Spectra were recorded prior to and after heating. The temperature did not influence the degradation since the absorption spectra did not change after heating.

### Spectrometer

In all studies the spectra were recorded by a Cary 4 spectrophotometer (Varian, USA). It is a two-beam spectrophotometer without integrating sphere, which measures the transmission by scanning over the wavelength range of interest. Without integrating sphere the measured absorbance includes also some scattered radiation. Therefore, the spectra of samples with inorganic filters, which scatter light, may show a too high absorbance.

### Area under the curve index (AUCI)

The AUC for UVA, UVA1 (340–400 nm), UVA2 (320–340 nm) and UVB was calculated for each spectrum before (AUC_before_) and after (AUC_after_) UVart (980 kJ/m^2 ^UVA and 12 kJ/m^2 ^of UV radiation (UVB included) and before and after UVnat. If the AUCI (AUCI = AUC_after_/AUC_before_) was >0.80, the sunscreen was considered photostable.

The AUC was calculated with the following equation:

∑λmin⁡λmax⁡A(λ)Δλ
 MathType@MTEF@5@5@+=feaafiart1ev1aaatCvAUfKttLearuWrP9MDH5MBPbIqV92AaeXatLxBI9gBaebbnrfifHhDYfgasaacH8akY=wiFfYdH8Gipec8Eeeu0xXdbba9frFj0=OqFfea0dXdd9vqai=hGuQ8kuc9pgc9s8qqaq=dirpe0xb9q8qiLsFr0=vr0=vr0dc8meaabaqaciaacaGaaeqabaqabeGadaaakeaadaaeWaqaaiabbgeabnaabmaabaacciGae83UdWgacaGLOaGaayzkaaGaeuiLdqKae83UdWgaleaacqWF7oaBcyGGTbqBcqGGPbqAcqGGUbGBaeaacqWF7oaBcyGGTbqBcqGGHbqycqGG4baEa0GaeyyeIuoaaaa@41B6@

where A is absorption and λ is wavelength. It was measured in steps of 1 nm.

For UVA λ_max _= 400 nm and λ_min _= 320 nm. The same calculation was done for each UV range respectively, before and after UVart and before and after UVnat.

Maier et al. used the difference between the spectral transmission before and after a defined UV exposure, ΔT. A product was labeled photounstable if the mean photoinstability was higher than 5% (1 mg/cm^2 ^product was used) [9]. In our study we chose the AUCI instead. Since we used 0.5 mg/cm^2 ^we considered the product photostable if the AUCI was higher than 0.8.

## Results

### Sunscreens

The photostability of the sunscreens tested varies considerably. The photounstable sunscreens start to degrade rather rapidly when exposed to the sun. After 30 min of UVnat, Sunscreens 1 and 3 are unstable (AUCI <0.80). Sunscreens containing inorganic chemical filters are more photostable in our study than sunscreens with organic chemical filters with the exception of Sunscreens 3 and 5

Sunscreens 5, 6 and 7 are photostable after UVnat; in the UVA range the AUCI was between 0.85 and 0.99 after 120 min and between 0.92 and 1.0 in the UVB range. Sunscreen 4 shows in the UVA range an AUCI of 0.87 after UVnat but 0.72 after UVart.

Sunscreens 1, 2 and 3 are unstable. They show after 90 min UVnat an AUCI between 0.41 and 0.76 in the UVA range and between 0.30 and 0.69 in the UVA1 range.

**Figure 1:**
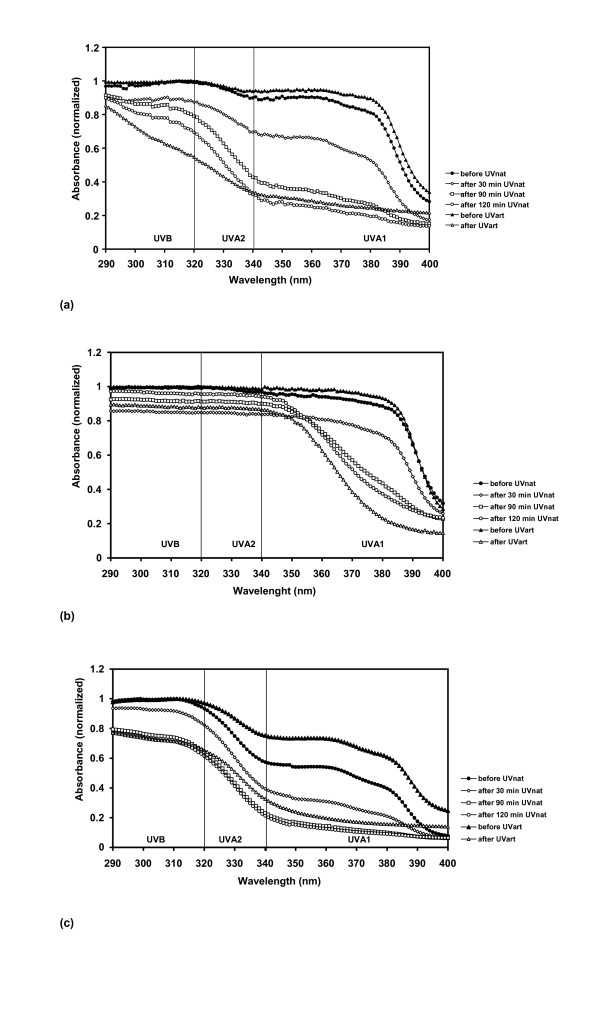
UV absorbance spectra of UVA **photounstable **sunscreens (AUCI <0.80). Before and after natural UV exposure (UVnat), and before and after artificial UV exposure (UVart). **(a) **Sunscreen 1 **(b) **Sunscreen 2 **(c) **Sunscreen 3.

Sunscreens 2, 4, 5, 6 and 7 are stable in the UVB region whereas Sunscreens 1 and 3 are not. During exposure, absorption ranges of Sunscreens 1, 2 and 3 are shifted towards shorter wavelengths (Fig. 1a–c).

In Table 3 the AUCI is presented.

**Figure 2:**
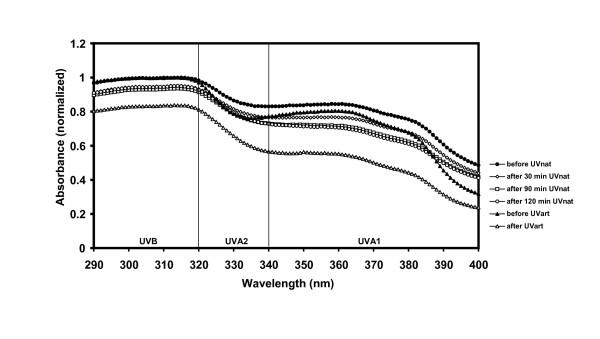
UV absorbance spectra of Sunscreen 4. Before and after natural UV exposure (UVnat), and before and after artificial UV exposure (UVart). Sunscreen 4 was photostable when exposed to natural UV in the UVA range but not to UVAart.

This is true for all three samples, after both UVnat and UVart. Sunscreen 4 is more unstable after UVart than to UVnat (Fig. 2).

The spectra were normalized by dividing the maximum value of the spectrum before irradiation by itself, so that the peak value of the spectrum before irradiation was set to 1.

**Figure 3:**
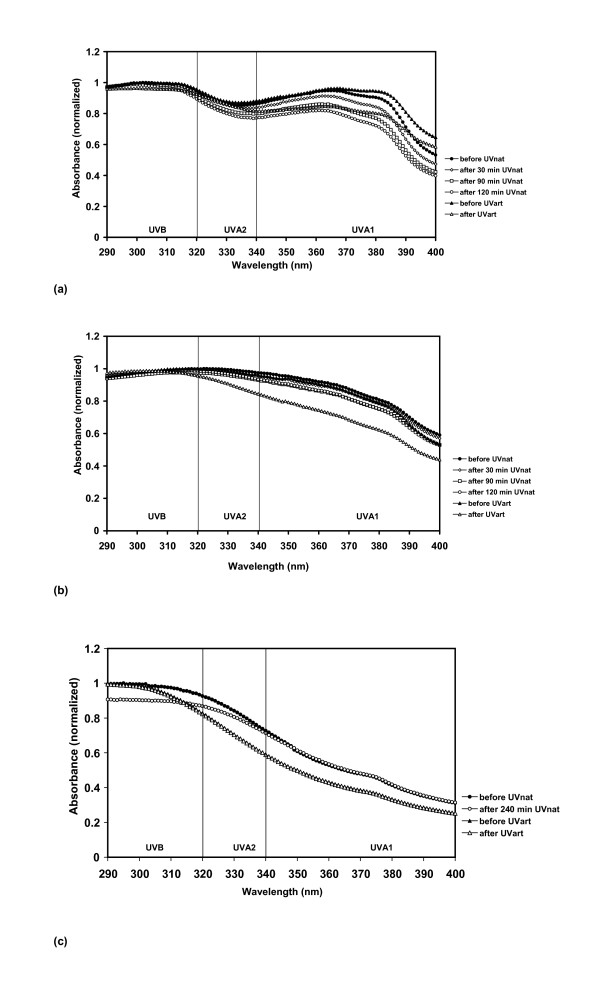
UV absorbance spectra of UVA **photostable **sunscreens (AUCI >0.80). Before and after natural UV exposure (UVnat), and before and after artificial UV exposure (UVart). **(a) **Sunscreen 5 **(b) **Sunscreen 6 **(c) **Sunscreen 7 was exposed to 240 min of UVnat.

The temperature was higher, during exposure to the UVart than during exposure to UVnat, but the temperature did not influence the absorption. Sunscreens 5, 6 and 7 were stable both after UVart and after UVnat. Sunscreens 6 and 7 were very little influenced by UV exposure (Fig. 3a–c). In agreement with findings from other studies, sunscreens with the UV filter combination ethylhexyl methoxycinnamate (EHMC) and butyl methoxydibenzoylmethane (BMDBM) were unstable [6,9,21,22].

## Discussion

In most cases UVnat compared to the UVart gave qualitatively similar results. However, UVnat, with a lower fluence rate than UVart, gave similar yields of degradation. In addition, the fluence rate of the UVAart was higher than that of the UVAnat, which could be expected to degrade the sunscreens faster. But this is not the case, except for Sunscreen 4. Since the dose of UVAart was higher than UVAnat, Sunscreen 4 probably provides sufficient protection for the consumer.

Commercial sunscreens generally have low viscosity in order to be easy to apply. The temperature increase of the samples during UV exposure, especially after UVart, may lower the viscosity further. This may result in reductions of the optical path lengths of the samples. However, this was not the case in our study since samples kept on a heating plate for 20 min at 50°C showed a similar spectrum before and after heating.

Four of the seven sunscreens contain TiO_2_. If the particles are too small they may lose their scattering effect and, consequently, not give as good protection as larger particles. This may be the case for Sunscreen 5 (Fig. 3a). Several other studies show that inorganic chemical filters are not always photostable [6,9-11]. Our study indicates the opposite, but seven sunscreens are a quite small amount of material, so this finding should be interpreted with caution.

When mixed with petrolatum, some sunscreens undergo degradation during exposure to UV radiation, especially in the UVA range [5]. This is also the case for one of the most frequently used UV filters BMDBM. This compound is included in six of the seven sunscreens studied here (Table 1). Our results confirm the findings from other studies that sunscreens containing the combination of EHMC and BMDBM are photounstable, regardless of what other UV filters they contain [6,9].

Some manufacturers of sunscreens claim that commercially available sunscreens are photostable because the photoactive species are in a vehicle that stabilizes them. This claim does not seem to be correct in several cases. There are several studies about how improvement of photostability may be obtained, e.g. with nanoparticle encapsulation of EHMC [23], liposphere preparation of BMDBM [24] or a combination with diethylhexyl syringylidene malonate and BMDBM [25]. These findings are very interesting and will hopefully lead to an improvement in photostability in commercial available products.

When sunscreens without metallic oxide particles are compared, Sunscreen 1 seems to be more rapidly degraded than BMDBM dissolved in petrolatum. Not only does the UVA protection decline after exposure, but also the UVB protection. EHMC is one of the two UVB-absorbing filters present in Sunscreen 2, and the only one in Sunscreen 1. EHMC dissolved in petrolatum is rather photounstable [5]. The vehicles of Sunscreens 1 and 2 are nearly identical. The UVA-absorbing compound benzophenone-3 (BZ-3) is added in Sunscreen 2. The presence of this compound may stabilize BMDBM, in agreement with earlier findings [26]. Another stabilizer that may work is anisotrizine (CAS no 187393-00-6)is [27]; however, that compound was not included in any of the products in this study. Sunscreen 2 also has a higher SPF. However, a degradation manifesting itself in the UVA1 region should be noted.

Sunscreen 5 is photostable but does not contain any metallic oxide particles. This may be due to a vehicle that successfully prevents degradation and/or due to microstructures of the emulsion itself (Fig. 3a). It is interesting to compare this spectrum with that of Sunscreen 3 (Fig. 1c) which, according to the list of contents, includes TiO_2 _particles but does not show the scattering slope. The size of the particles may be too small (15 nm, according to the producer) to influence the absorption spectrum in the visible range. Small particles of TiO_2 _are expected to give maximal scattering in the UVB or UVC region. Larger particles can cause significant scattering also in the UVA and visible region. It follows that the small nanoparticles cannot give good protection in the UVA region in this case.

The peak between 350 and 375 nm in the absorption spectra of Sunscreens 3, 4 and 5 (Figs. 1c, 2, 3a) can be attributed to BMDBM. In view of this it should be noted that the UV exposure makes the products react quite differently. In Sunscreen 3 the BMDBM peak almost vanishes totally after 30 min of UVnat, while the peaks in the other two sunscreens are more stable. We suggested above why there could be degradation in the UVA range in Sunscreen 3 despite the presence of TiO_2 _particles. The reported stabilizing effect of 4-Methylbenzylidene camphor (MBC) [26] does not manifest itself in the case of Sunscreen 3.

The photostable Sunscreen 6 contains, in addition to BMDBM and TiO_2_, a third UVA absorber, terephthalylidene dicamphor sulfonic acid (TLDCSA), which can stabilize BMDBM. It has also been shown that TiO_2 _may stabilize ketoprofen and may be used in protecting photounstable species [28].

Many commercial sunscreens give, according to the manufacturers, good UVA and UVB protection. However, the photostability of the sunscreen in the UVA range is not always adequate. Most sunscreens offer good protection against UVB while the UVA photostability of some products decreases substantially during UV exposure. The potential toxicity of the photoproducts also needs to be investigated further.

For the consumer it is very difficult to know what product to choose, since the photostability varies between different brands and the photostability is not marked on the bottle. To know which photoactive compound the sunscreen contains is not good enough. The stability also depends on factors like preservatives, oxygen radical scavengers, and base formulation. It is not reasonable that the ordinary consumer should have knowledge of this. If the product claims to give broadband protection, this protection should remain also after sun exposure. The fact that sunscreens are photounstable has been known for many years. Our study clearly shows that there are still many photounstable products on the market. When buying a sunscreen, the consumer should automatically receive a photostable product.

## Conclusion

The present study shows that several commercially available sunscreens are not photo stable. Degradation is clearly manifested in the absorption region in the UVA range after solar irradiation. In general, sunscreens with TiO_2 _particles seem to be more photostable, with Sunscreens 3 and 5 as exceptions. Special focus should be on the commonly used UVA absorber BMDBM. In three out of six sunscreens in our study this molecule was degraded during UV exposure. Stabilizers of BMDBM may work, but not under all conditions. There is a need for a standardized method to measure photostability and the photostability should be marked on the sunscreen product.

## Competing interests

The author(s) declare that they have no competing interests.

## Authors' contributions

HG and NT-W have made contributions to conception of design and interpretation of the data. They carried out the experimental set-up during the absorption studies and wrote the main part of the manuscript.

BS carried out some of the absorption studies and drafted the manuscript.

AR have made substantial contributions to conception of design, interpretation of the data and drafting and revising the manuscript. AR also participated in the coordination of the study.

AJ, JM, OL and A-MW have made substantial contribution to concept of design and drafting and revising the manuscript critically.

All authors read and approved the final manuscript.

## Pre-publication history

The pre-publication history for this paper can be accessed here:



## Figures and Tables

**Table 1:** The photoactive compounds in the investigated sunscreens, CAS no and SPF of the product.

**Photoactive compound**	**CAS no**	**Mainly protection against**		**Active ingredients in the seven investigated sunscreen products**						
		**UVA**	**UVB**	**1**	**2**	**3**	**4**	**5**	**6**	**7**

**EHMC**	5466-77-3		x	x	x	x				
**MBC**	36861-47-9		x			x	x	x		
**EHT**	88122-99-0		x				x			
**OC**	6197-30-4		x						x	
**BMDBM**	70356-09-1	x		x	x	x	x	x	x	
**BZ-3**	131-57-7	x			x	x				
**TLDCSA**	90457-82-2	x							x	
**TiO**_2_	13463-67-7		x			x	x		x	x
**ZnO**	1314-13-2	x								x

**SPF**				4	14	10	10	6	10	15

**CAS **Chemical Abstracts Service

**EHMC **ethylhexyl methoxycinnamate **MBC **4-methylbenzylidene camphor

**EHT **ethylhexyl triazone **OC **octocrylene **BMDBM **butyl methoxydibenzoylmethane

**BZ-3 **benzophenone-3 **TLDCSA **terephthalylidene dicamphor sulfonic acid

**TiO**_2_titanium dioxide **ZnO **zinc oxide

**SPF **Sun Protection Factor

**Table 2:** The dose of natural UV radiation the investigated sunscreens received

**Sunscreen**	**Exposure time (min)**	**UVA radiation (kJ/m**^2^**)**	**Erythemal effective radiation (J/m**^2^**)**	**SED**
**1**	30	54	**	
	90	180	**	
	120	260	**	
**2**	30	54	100	1
	90	180	410	4.1
	120	260	610	6.1
**3**	30	65	140	1.4
	90	210	530	5.3
	120	280	730	7.3
**4**	30	65	**	
	90	210	**	
	120	280	**	
**5**	30	**	**	
	90	**	**	
	120	210	**	
**6**	30	47	140	1.4
	90	140	300	3.0
	120	210	370	3.7
**7**	30	65	150	1.5
	90	130	320	3.2
	240*	570	1500	15

Data from the Swedish Metrological and Hydrological Institute (SMHI)

* The exposure time for Sunscreen 7 it was 30 min, 90 min and 240 min due to technical obstacles

** Data not available due to technical obstacles at SMHI.

**Table 3:** Summary of the AUCI values for the investigated sunscreens

	**After natural UV exposure**												**After artificial UV exposure**			
**Sunscreen**	**UVA**			**UVA1**			**UVA2**			**UVB**			**UVA**	**UVA1**	**UVA2**	**UVB**

	**30 min**	**90 min**	**120 min**	**30 min**	**90 min**	**120 min**	**30 min**	**90 min**	**120 min**	**30 min**	**90 min**	**120 min**				

**1**	**0.72**	**0.46**	**0.36**	**0.69**	**0.38**	**0.29**	0.83	**0.65**	**0.54**	0.91	0.87	0.81	**0.36**	**0.32**	**0.45**	**0.69**
**2**	0.84	**0.76**	**0.75**	0.83	**0.69**	**0.67**	0.86	0.92	0.97	0.86	0.92	0.97	**0.63**	**0.53**	0.88	0.89
**3**	**0.67**	**0.41**	**0.41**	**0.59**	**0.30**	**0.34**	0.81	**0.58**	**0.52**	0.92	**0.75**	**0.63**	**0.40**	**0.31**	**0.58**	**0.73**
**4**	0.92	0.86	0.87	0.91	0.85	0.83	0.94	0.91	0.91	0.95	0.93	0.95	**0.72**	**0.69**	0.81	0.83
**5**	0.96	0.89	0.85	0.94	0.87	0.83	0.99	0.95	0.93	0.99	0.99	0.98	0.90	0.88	0.97	0.97
**6**	0.98	0.94	0.94	0.97	0.93	0.93	0.98	0.96	0.97	0.99	0.99	1.00	0.85	0.82	0.92	1.00

**7**			0.99*			1.00*			0.96*			0.92*	0.99	0.99	1.00	0.99

The AUCI is defined as AUC_after_/AUC_before_. The bold numbers show when AUCI is <0.80.

* Sunscreen 7 was exposed to natural UV during 240 min.
